# Facial nodules following filler injections after initiating compounded tirzepatide use

**DOI:** 10.1016/j.jdcr.2026.03.042

**Published:** 2026-03-27

**Authors:** Moore Luke, Hurley Kara, Moore Angela

**Affiliations:** a Arlington Center for Dermatology, Arlington, Texas; b Arlington Research Center, Arlington, Texas; c McGovern Medical School at UTHealth Houston, Houston, Texas; d Department of Dermatology, Texas Tech University Health Sciences Center, Lubbock, Texas; e Texas Christian University Anne Burnett Marion School of Medicine, Fort Worth, Texas; f Baylor University Medical Center, Dallas, Texas

**Keywords:** botulinum toxin, calcium hydroxyapatite, compounded tirzepatide, filler nodules, GLP-1, GLP-1 receptor agonist, hyaluronic acid

## Introduction

Glucagon-like peptide-1 (GLP-1) was identified in the 1980s as a transient molecule that promotes insulin secretion before being rapidly degraded within a minute.^1^ More recently, longer-lasting glucagon-like peptide-1 receptor agonists (GLP-1RAs) were developed. The first GLP-RA, exenatide, was approved in 2005 for the treatment of type 2 diabetes and has a half-life of approximately 2.5 hours; since 2014, GLP-1RAs have been Food and Drug Administration–approved for weight loss.^1^ GLP-1RAs mimic receptors responsible for increasing insulin secretion, decreasing glucagon release, slowing gastric emptying, and reducing appetite, which leads to the net effect of better-controlled serum glucose levels and weight loss.^1^^,^^2^ While most GLP-1RAs target the GLP-1 receptor, tirzepatide activates both the GLP-1 and the glucose-dependent insulinotropic polypeptide (GIP) receptors. GLP-1 and GIP receptors both function through incretin hormones released by enteroendocrine cells, although they are located in different parts of the gastrointestinal tract.^3^ Newer GLP-RAs have been developed with less frequent dosing, with tirzepatide being injected weekly^3^^,^^4^ with a half-life of roughly 5 days.^1^

Tirzepatide prescriptions have soared due to the high incidence of type 2 diabetes and obesity. The rate of compounded tirzepatide also increased, particularly during the shortage of the branded tirzepatide from December 2022 to March 2025. Here, we report novel reactions to filler injections, which occurred after the patient starting using compounded tirzepatide.

## Case report

A 66-year-old female presented with 9 months of persistent dermal nodules at sites of hyaluronic acid and calcium hydroxyapatite filler injection sites as well as at incobotoxulinumtoxin A injection sites. She had a history of allergic rhinitis and recurrent sinusitis and no tobacco, alcohol, or drug use. She had received similar filler and incobotoxulinumtoxin A injections without any dermal nodules from different aestheticians on multiple occasions prior to initiating compounded tirzepatide.

**Fig 1:**
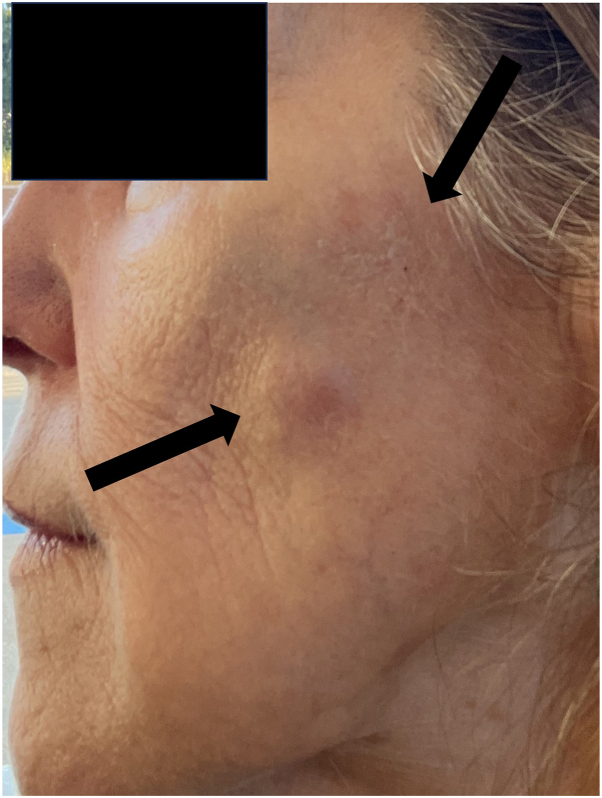
Left mid-cheek and left zygomatic arch with 2.5 cm erythematous nodules at the site of calcium hydroxyapatite filler injection.

She had been first prescribed compounded tirzepatide 2.5 mg weekly for weight loss 11 months prior to presentation. She underwent more injections with calcium hydroxyapatite injections 1.5 mL at the cheeks, hyaluronic acid 1 mL at the lips, and 27 units of incobotulinumtoxin A 1 month after initiating compounded tirzepatide. An initial large nodule on the left cheek with a smaller nodule at the left zygomatic arch suddenly appeared several weeks after these injections (Fig 1).

**Fig 2:**
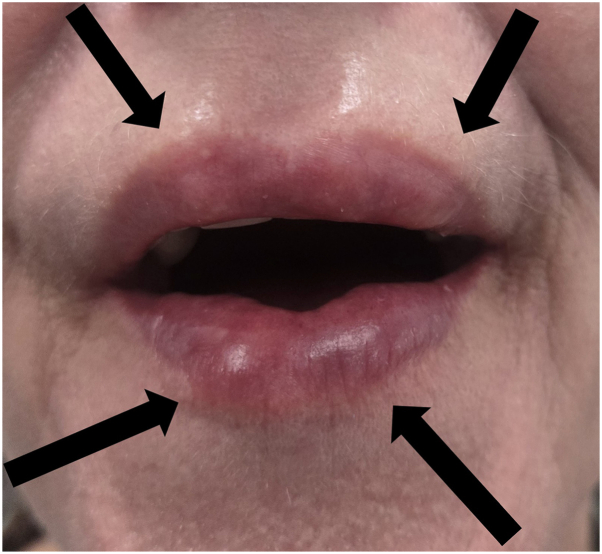
Upper and lower lips with 8 mm to 1 cm mobile nodules at the site of hyaluronic acid filler injections.

**Fig 3:**
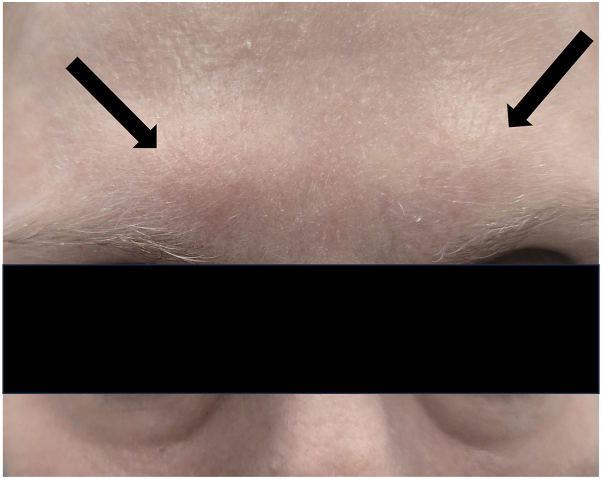
Glabella with 1.2 cm mobile nodules at sites of incobotulinumtoxin A injection.

The nodules were refractory to 0.5 mL intralesional triamcinolone 5 mg/mL and hyaluronidase injections by the outside practitioner; instead, the left cheek site became depressed. The compounded tirzepatide dosing was titrated down to 0.5 mg weekly over the next several weeks as the patient was satisfied with her weight loss but wanted to stay on tirzepatide for weight maintenance. Further injections of 1 mL of hyaluronic acid at the temples and marionette lines and 1.5 mL of calcium hydroxyapatite at the cheeks, performed by different practitioners, were done in attempt to mask the original nodules. However, these attempts only resulted in the formation of new, persistent small nodules (Fig 2) despite the use of a 1 mL vial of intralesional hyaluronidase. Small nodules subsequently formed at the glabella at the site of prior incobotulinumtoxin A injections (Fig 3).

As nodules continued to form, the patient completed 2 30-day courses of twice daily doxycycline 100 mg over 4 months with no improvement. When the patient presented with these persistent facial nodules refractory to oral antibiotics and hyaluronidase, compounded tirzepatide was proposed to contribute to their formation, and subsequently discontinued. The facial nodules did not resolve immediately but no new nodules appeared subsequently at sites of prior filler and botulinumtoxin injections.

Isotretinoin 20 mg daily was initiated, and the lesions resolved within 1 month. A rechallenge of 2.5 mg of compounded tirzepatide was started while on isotretinoin, and no nodules appeared after hyaluronic acid injections with 2 mL to the lateral cheekbones, 1 mL to the medial cheekbones, and 1 mL to the lips and marionette lines.

One month later, the dosage of compounded tirzepatide was increased to 5 mg for 3 weeks and subsequently increased to 7.5 mg while still taking isotretinoin 20 mg daily. Two 2-mm nodules subsequently formed on the upper lip at the hyaluronic injection sites 4 weeks later.

## Discussion

With increased utilization, several cutaneous skin manifestations of GLP-1 agonists have been reported, including bullous pemphigoid, angioedema,^2^ eosinophilic fasciitis, drug-induced lupus,^5^ and leukocytoclastic vasculitis.^6^ This case report highlights facial dermal nodules at the sites of filler and incobotulinumtoxin A injections, a previously unreported association to the GLP-1A and GIP agonist tirzepatide. Since new facial nodules appeared after starting compounded tirzepatide, continued forming during tirzepatide use, ceased forming after stopping compounded tirzepatide, and started reforming upon restarting tirzepatide use, a temporal association of tirzepatide with facial nodules exists.

The facial nodules likely result from an adverse reaction instead of the injection itself as the hyaluronic acid filler injections did not respond to hyaluronidase injections or doxycycline. Due to the nodules appearing on the face, biopsy of the lesions was not taken. While infectious etiologies cannot be ruled out, the numerous locations and appearance of nodules over several months following injections from various clinics would suggest a reaction to compounded tirzepatide injection itself. The resolution of the nodules after starting isotretinoin strengthens an inflammatory cause behind the nodules.

The rechallenge of compounded tirzepatide further supports compounded tirzepatide as an associated agent behind the nodules as they did not occur upon cession of tirzepatide. The increased dosing of tirzepatide required to elicit the nodules might result from the protective effects of isotretinoin. Limitations of this case study include unknown ingredients in the compounded tirzepatide and a lack of biopsy of nodules and the inability to draw causative conclusions. Due to the overlap in demographics between patients receiving GLP-1RAs and those seeking filler or botulinum toxin injections, dermatologists and other practitioners should be aware of this potential association to compounded tirzepatide. Further research and evidence into the possible association between compounded tirzepatide and filler nodules before is warranted to highlight whether the tirzepatide, compounded ingredients, or different etiologies altogether were responsible.

### Declaration of generative AI and AI-assisted technologies in the writing process

No generative AI was used in the manuscript composition.

## Conflicts of interest

None disclosed.
